# Inhibition of lipopolysaccharide-induced nitric oxide and prostaglandin E_2_ production by chloroform fraction of *Cudrania tricuspidata* in RAW 264.7 macrophages

**DOI:** 10.1186/1472-6882-12-250

**Published:** 2012-12-10

**Authors:** Gabsik Yang, Kyungjin Lee, Mihwa Lee, Inhye Ham, Ho-Young Choi

**Affiliations:** 1 Department of Herbology, College of Oriental Medicine, Kyung Hee University, 1 Hoegi-Dong, Seoul, Dongdaemun-Gu, Republic of Korea

**Keywords:** *Cudrania tricuspidata*, Nitric oxide, Prostaglandin E_2_, Lipopolysaccharide CHCl_3_ partitioned methanol extract of *Cudrania tricuspidata*, CTC, Lipopolysaccharide, LPS, prostaglandin E_2_, PGE_2,_ nitric oxide, NO

## Abstract

**Background:**

*Cudrania tricuspidata* extract is an important traditional herbal remedy for tumors, inflammation, gastritis, and liver damage and is predominantly used in Korea, China, and Japan. However, the anti-inflammatory effects of the extract have not yet been conclusively proved.

**Methods:**

In this study, we investigated the effects of the CHCl_3_ fraction (CTC) of a methanol extract of *C. tricuspidata* on the lipopolysaccharide (LPS)-induced nitric oxide (NO) and prostaglandin E2 (PGE2) production in RAW 264.7 macrophage cells and mouse peritoneal macrophages, and the levels of pro-inflammatory cytokines tumor necrosis factor-α (TNF-α), interleukin (IL)-1β and IL-6 in RAW 264.7 macrophage cells.

**Results:**

We observed that the protein expression levels of inducible NO synthase and COX-2 enzymes were markedly inhibited by CTC in a concentration-dependent manner. In addition, CTC reduced the production of TNF-α, IL-1β, and IL-6 in the LPS-stimulated RAW 264.7 macrophage cells.

**Conclusions:**

Our results show that the *C. tricuspidata* extract could modulate macrophage-mediated inflammatory functions such as the overproduction of cytokines, NO, and PGE2. The CTC was found to be the active fraction in this context.

## Background

Inflammation is the result of the host’s immune response to pathogenic challenges or tissue injuries, and under normal conditions, tissue structure and function are restored to normal when the response ends. Moreover, normal inflammatory responses are self-limited by a process that involves the downregulation of pro-inflammatory proteins and the upregulation of anti-inflammatory proteins [1]. Thus, acute inflammation is essentially a beneficial process, particularly in response to infectious pathogens, whereas chronic inflammation is an undesirable phenomenon that can lead to inflammatory diseases [2].

Macrophages are extraordinarily versatile cells, and the ability of the macrophages to exploit their full functional repertoire is essential for host immunity [3]. Macrophages detect pathogenic substances through pattern-recognition receptors and subsequently initiate and regulate inflammatory responses [3] by using a wide range of soluble pro-inflammatory mediators. Lipopolysaccharide (LPS) is one of the most powerful activators of macrophages, and macrophages and monocytes that have been activated by LPS are known to produce inflammatory mediators such as nitric oxide (NO) and other free radicals, in addition to numerous cytokines such as tumor necrosis factor-α (TNF-α), interleukin (IL)-1β, and IL-6 [4-6]. Bacterial pathogens, like LPS, activate cytokine networks by inducing many pro-inflammatory genes. Moreover, these inductions are mediated via the activation of inducible transcription factors [7]. During the inflammatory process, large amounts of the pro-inflammatory mediators NO and prostaglandin E2 (PGE2) are generated by the inducible isoforms of NO synthase (NOS) and cyclooxygenase-2 (COX-2) [8]. In mammalian cells, NO is synthesized by 3 isoforms of NOS, namely, neuronal NOS (nNOS), endothelial NOS (eNOS), and inducible NOS (iNOS). Although nNOS and eNOS are constitutively expressed, iNOS is specifically expressed in response to interferon-γ (IFN-γ), LPS, and various pro-inflammatory cytokines [9,10]. Several studies have suggested that the overexpression of COX-2 or iNOS is intimately involved in the pathogenesis of inflammation, cancer, multiple sclerosis, Parkinson’s syndrome, Alzheimer’s disease [11], and other diseases. Thus, many efforts have been made to develop enzyme inhibitors or repressors of enzyme formation that selectively target the inducible forms of these enzymes and, in particular, do not affect the activities of their respective constitutive isoforms. Therefore, inhibiting the production of NO and PGE2 is an important therapeutic target in the development of anti-inflammatory agents.

*Cudrania tricuspidata* (CT) is a deciduous tree distributed across South Korea, China, and Japan, and the cortex and root bark of CT have been frequently used as a traditional medicine to treat inflammation and tumors [12]. Several previous reports have described various effects of the CT extract, which include antioxidant activity [13], inhibitory effect on NOS [14], and effects on the proliferation of inflammatory immune cells and tumor cells [15,16]. However, no studies to date have investigated the effects of the CHCl_3_ fraction (CTC) of the methanol extract of CT on inflammatory reactions.

In the present study, we show that CTC reduces the production of NO, PGE2, and TNF-α in RAW 264.7 cells and mouse macrophage cells when these cells are treated with recombinant LPS.

## Methods

### Plant materials

Non-cultivated CT was collected from the Chiri Mountain in the far south of the Republic of Korea. Collection was made on February 20, 2007, a time when the plants have many thorns and few leaves. The plant materials were identified by Professor Ho-Young Choi at the Herbology Laboratory, College of Oriental Medicine, Kyunghee University, Seoul. Fresh CT was removed using a sickle and dried in a dark, well-ventilated place. Air-dried CT (1 kg) was cut into pieces by using a fodder chopper, and the extract was prepared by incubating with 80% methanol at room temperature for several days. Then the CT methanol extract (CTM) was fractionated using CHCl_3_, and the CHCl_3_ extracts were evaporated to afford a CHCl_3_-soluble (14 g) fraction (CTC), which was dissolved in DMSO (Sigma, St. Louis, MO, USA) to obtain a final concentration of 100 mg/ml, and was stored at −20°C until required. To use, the extract was further diluted to different concentrations by using cell culture media (DMSO < 0.1%).

### Animals

Male BALB/c mice (6–8 weeks of age) were purchased from the Korean branch of Taconic, Samtaco (Osan, Korea). Mice were kept in a temperature- and humidity-controlled, pathogen-free animal facility at Kyung Hee University and provided standard mouse chow and water *ad libitum*. All animal care and experimental procedures were complied with university guidelines of by Korean National Veterinary Research and Quarantine Service, World Organization of Animal Health (WOAH) and were approved by the Ethical Committee for Animal Care and the Use of laboratory animals Kyung Hee University (Approval number # KHUASP(SE)-11-039).

### Cell culture and sample treatment

RAW 264.7 cells were obtained from the Korean Cell Line Bank (Seoul), and were grown at 37°C in DMEM medium supplemented with 10% FBS, penicillin (100 units/ml), and streptomycin sulfate (100 mg/ml) in a humidified 5% CO_2_ atmosphere. Cells were incubated with CT at increasing concentrations (62, 125, 250, and 500 mg/ml), and then stimulated with LPS (1 or 10 μg/ml) for the indicated times.

### Macrophage isolation and culture

Mice were injected intraperitoneally (*i.p.*) with 2 ml of sterile thioglycollate medium (Becton Dickinson, Franklin Lakes, NJ, USA). Three days later, macrophages were collected by peritoneal lavage with cold Dulbecco’s modified Eagle’s medium (DMEM). After centrifugation of the lavage, red blood cells were removed by lysis for 3 min in lysis buffer (150 mM NH_4_Cl, 1 mM KHCO_3_, and 0.1 mM Na_2_EDTA). After centrifugation, the cells were resuspended in DMEM supplemented with 1% antibiotic and antimycotic solution (Invitrogen, Carlsbad, CA, USA) and 10% fetal bovine serum (WeIGENE, Deagu, Korea), then incubated for 90 min in a humidified atmosphere of 5% CO_2_ at 37°C. Non-adherent cells were removed by washing, and the adherent cells were harvested and seeded for the various assays.

### MTS-tetrazolium salt assay

Cell viability was measured based on the formation of blue formazan metabolized from colorless MTS by mitochondrial dehydrogenases, which are active only in live cells. RAW 264.7 macrophages were plated in 96-well plates at a density of 4 × 10^3^ cells per well for 24 h, and then washed. Cells incubated with various concentrations of CTM and CTC were treated with LPS (*E*. *coli* 026:B6; Sigma, MO, USA) for 24 h and then incubated in 0.5 mg/ml MTS solution. Viabilities were determined using colorimetric MTS assay*.*

### Determinations of nitrite concentrations

RAW 264.7 cells were plated at 2.5 × 10^5^ cells/ml in 24 well-plates and then incubated with or without LPS (1 μg/ml) in the absence or presence of various concentrations (62, 125, 250 and 500 μg/ml) of CTC for 24 h. The nitrite accumulated in culture medium was measured as an indicated of NO production based on the Griess reaction. Briefly, 100 μl of cell culture medium was mixed with 100 μl of Griess reagent [equal volumes of 1% (w/v) sulfanilamide in 5% (v/v) phosphoric acid and 0.1% (w/v) naphtylethylenediamine-HCl], incubated at room temperature for 10 min, and then the absorbance at 540 nm was measured in a microplate reader (VersaMax™, Molecular Device, USA). Fresh culture medium was used as the blank in all experiments. The amount of nitrite in the samples was measured with the serial dilution standard curve of sodium nitrite.

### Western blot analysis

Cellular proteins were extracted from control and CT-treated RAW 264.7 cells. Briefly, cells were collected by centrifugation and the pellets so obtained were washed once with phosphate-buffered saline (PBS). Pellets were then resuspended in extraction lysis buffer (50 mM HEPES pH 7.0, 250 mM NaCl, 5 mM EDTA, 0.1% Nonidet P-40, 1 mM phenylmethylsulfonyl fluoride, 0.5 mM dithiothreitol, 5 mM Na fluoride and 0.5 mM Na orthovanadate) containing 5 mg/ml leupeptin and 5 mg/ml aprotinin and incubated for 20 min at 4°C. Cell debris was removed by microcentrifugation, and supernatants were rapidly frozen. Protein concentrations were determined using the Bio-Rad protein assay reagent **(**Hercules, CA), according to the manufacturer’s instructions. Proteins (40 μg) were separated by 10% SDS-polyacrylamide gel electrophoresis, and electroblotted onto nitrocellulose membranes. Immunoblots were incubated overnight with blocking solution (5% skimmed milk) at 4°C, and then for 4 h with a primary antibody. Blots were then washed three times with Tween 20/Tris-buffered saline (TBST), incubated in 1:100 alkaline phosphatase-conjugated secondary antibody for 1 h at room temperature, washed three times with TBST, and finally reacted with BCIP-NBT solution (Nakanai Tesque, Japan). The monoclonal antibodies (iNOS, COX-2 and β-actin), and the peroxidase-conjugated secondary antibody were purchased from Santa Cruz Biotechnology, Inc. (Santa Cruz, CA, USA). Density ratios versus β-actin were determined by densitometry analysis (Scion NIH Image 1.63).

### Measurement of PGE_2_ levels

PGE_2_ levels in culture media were analyzed using PGE_2_ assay kits (Parameter^TM^; R&D Systems, MN, USA). The PGE_2_ standard and the RD5-39 in the kits were used to construct a standard curve. Briefly, 100 μl of the medium was mixed with 50 μl of primary antibody solution and PGE_2_ conjugate and incubated for 2 h at room temperature on a shaker. Cells were then washed out of wells using 400 μl of 1× washing buffer; then, color reagent (200 μl) was added and, 30 min later, stop solution (50 μl) was added and mixed in. Absorbance was measured at 450/570 nm using a VersaMax^TM^ microplate reader (Molecular Devices, CA, USA).

### Cytokine array assays

Mouse cytokine antibody array membranes coated with 40 specific cytokine antibodies (RayBioTM Mouse Inflammation Antibody Array 1, RayBiotech, Inc.) were probed with isolated total protein samples. The membranes were blocked by incubation in blocking buffer for 30 min at room temperature and then incubated with samples for 1 h at room temperature. Membranes were then washed three times with Wash Buffer I and twice with Wash Buffer II for 5 min at room temperature per wash and then incubated with biotin-conjugated antibodies at 4°C overnight. Membranes were then washed, incubated with HRP-conjugated streptavidin for 1 h at room temperature, incubated with detection buffer for 1 min, and exposed to X-ray film for 40 s (Kodak Inc.). Exposed films were digitized and relative cytokine levels were determined by densitometry (Scion NIH Image 1.63), by subtracting background staining level and normalizing to positive control spots, which were used as internal standards.

### Statistical analysis

The results shown were obtained from at least three independent experiments and are presented as means ± SDs. Statistical analyses were performed by one-way analysis of variance (ANOVA) with Tukey’s and Duncan’s post hoc tests. All statistical analyses were performed using SPSS v12.0 software. *P* values < 0.05 were considered to indicate statistical significance.

## Results

### Effects of CTC on the viability of RAW 265.7 cells

**Figure 1:**
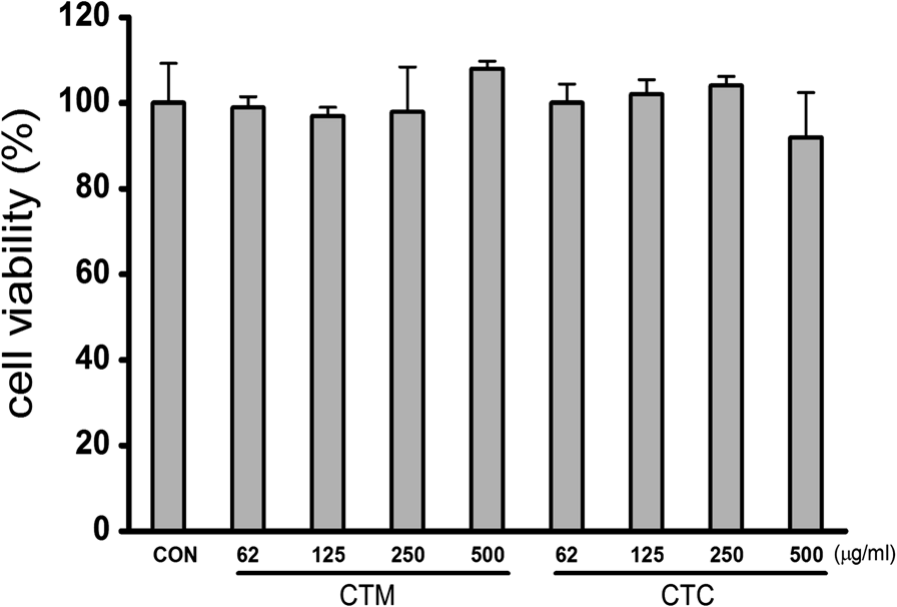
**Effect of CTM on the viability of LPS-stimulated RAW 264.7 cells. **Cells (5 × 10^4^) were incubated with CTM and CTC (62, 125, 250, 500 μg/ml) at 37°C for 24 h. Cell viabilities were evaluated using MTS assays. Data represent the means ± SDs of three independent experiments.

We examined the effects of CTM on the viability of RAW 264.7 cells *in vitro* by incubating cells with 62 μg/ml, 125 μg/ml, 250 μg/ml, or 500 μg/ml of CT or CTC for 24 h. The results of the MTS assay showed that CT and CTC were not cytotoxic to RAW 264.7 cells at concentrations up to 500 μg/ml (Figure 1). 

### Effects of CTC on LPS-induced NO and PGE2 production

**Figure 2:**
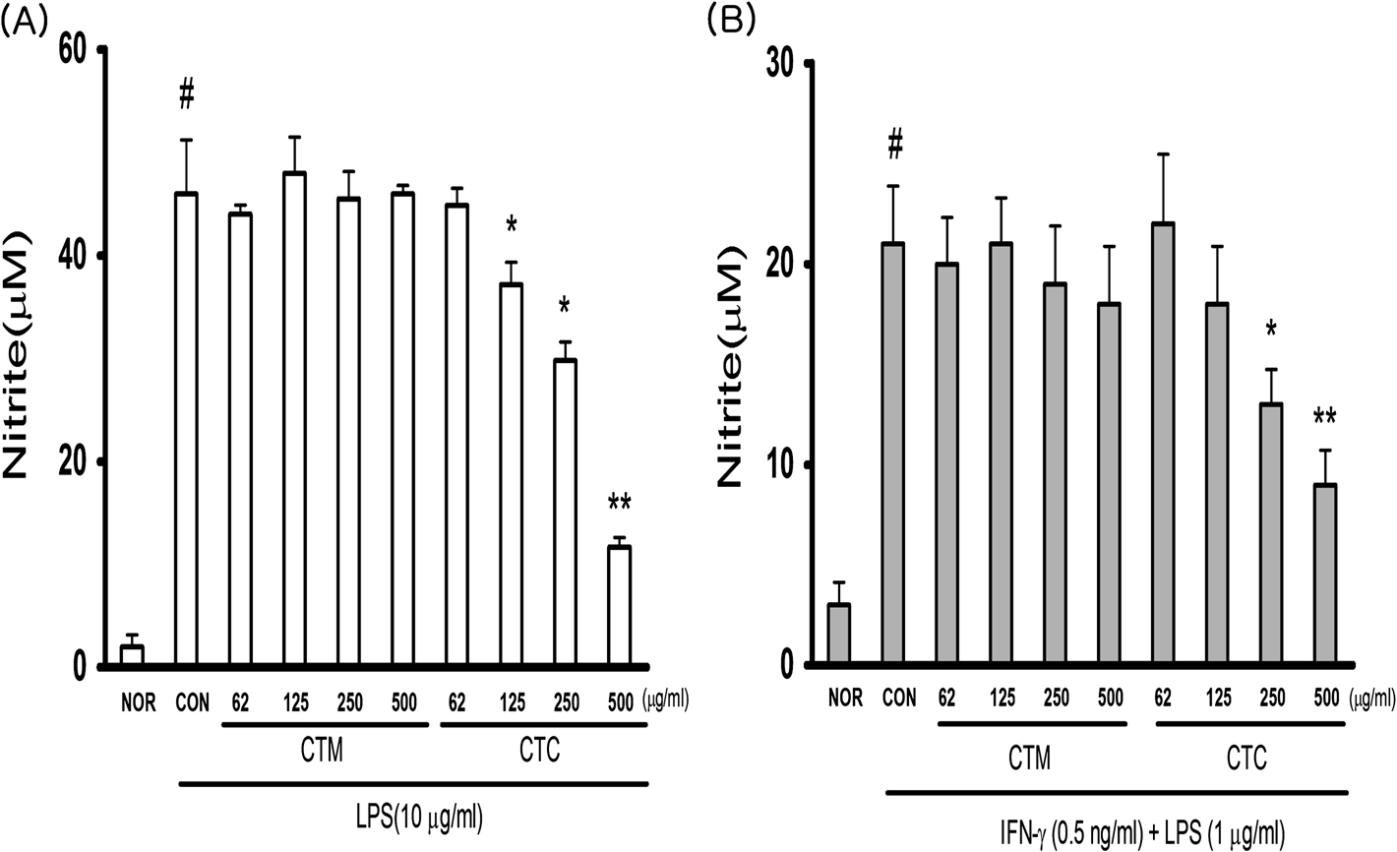
**Effect of CTM on LPS-induced NO levels in RAW 264.7 cells. **(**A**) Cells were treated with different concentrations of CTM and CTC (62, 125, 250, 500 μg/ml) for 1 h, then with LPS (10 μg/ml) for 24 h. Culture supernatants were subsequently isolated and analyzed for nitrite levels. (**B**) Nitrite levels in culture supernatants were measured after macrophages (2 × 10^6^ cells/ml) were primed with IFN-γ (0.5 ng/ml) for 2 h and then stimulated with LPS (1 μg/ml) in the presence of CTM and CTC for 18 h. Values are means ± SDs of three separate experiments. (^#^*p* < 0.05 vs. the non-treated control group; *p < 0.05, **p < 0.01 vs. the LPS-treated group).

**Figure 3:**
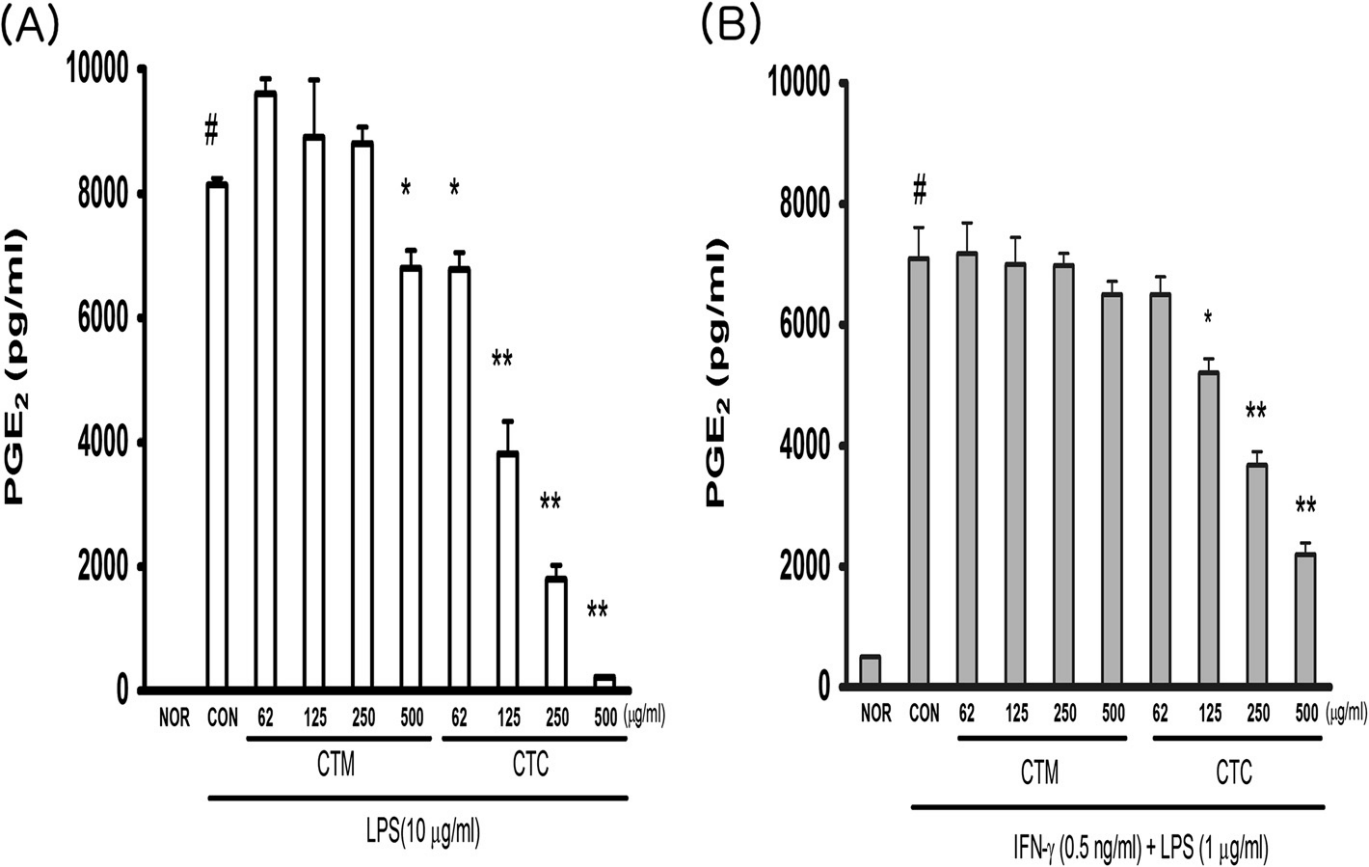
**Effect of CTM on LPS-induced PGE**_**2 **_**production in RAW 264.7 cells. **(**A**) PGE_2_ production was measured in RAW 264.7 cells treated with different concentrations of CTM and CTC (62, 125, 250, 500 μg/ml) for 1 h, then incubated with LPS (10 μg/ml) for 24 h. (**B**) PGE_2_ production in culture supernatants was measured after macrophages (2 × 10^6^ cells/ml) were primed with IFN-γ (0.5 ng/ml) for 2 h and then stimulated with LPS (1 μg/ml) in the presence of CTM and CTC for 18 h. Values are means ± SDs of three separate experiments. (^#^*p* < 0.05 vs. the non-treated control group; ^*^p < 0.05, **p < 0.01 vs. the LPS-treated group).

To investigate the anti-inflammatory properties of CTC, we used RAW 264.7 cells, which produce NO and PGE2 when stimulated by LPS. The cells were pre-incubated with varying concentrations of CTM and CTC (each at 62 μg/ml, 125 μg/ml, 250 μg/ml, and 500 μg/ml) for 1 h and then stimulated with 10 μg/ml LPS for 24 h. LPS and CT were not added to the controls (Con). The nitrite and PGE2 levels in the media were determined, and we observed that CTC reduced NO and PGE2 production in a dose-dependent manner (Figures 2A and 3A). To examine the potential effects of CTC on LPS-induced NO and PGE2 production in macrophages, the cells were pretreated with IFN-γ (0.5 ng/ml) for 2 h and then stimulated with LPS (1 μg/ml) and varying concentrations of either CTM or CTC (62 μg/ml, 125 μg/ml, 250 μg/ml, and 500 μg/ml each) for 18 h. The NO and PGE2 concentrations in the culture supernatants were suppressed by CTC in a dose-dependent manner (Figures 2B and 3B). 

### Effects of CTC on LPS-induced iNOS and COX-2 protein Levels

**Figure 4:**
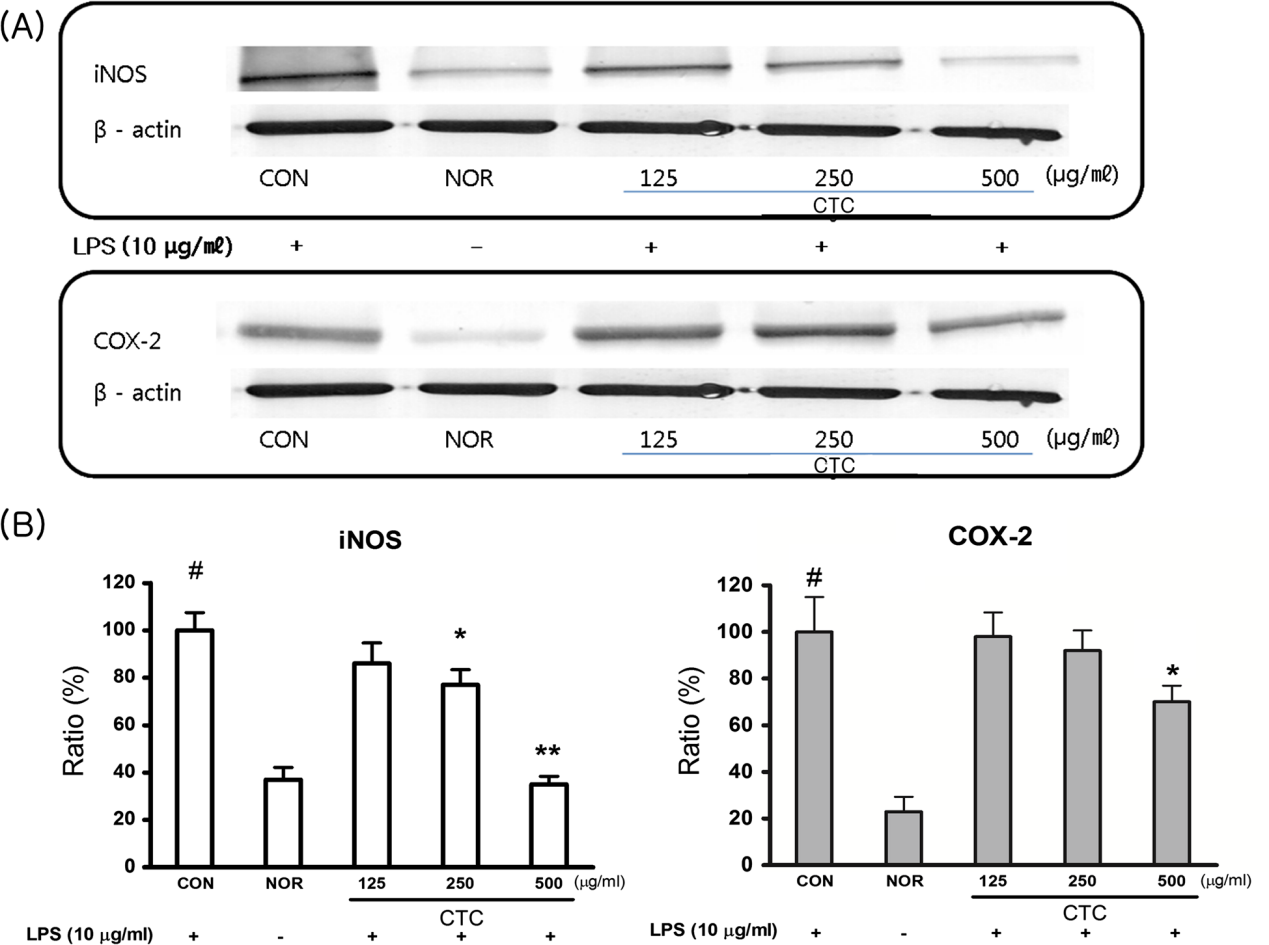
**Effect of CTC on LPS-induced iNOS and COX-2 protein levels in RAW 264.7 cells. **(**A**) Cells were treated with different concentrations of CTC (125, 250, 500 μg/ml) for 1 h and then with LPS (10 μg/ml) for 24 h. β-actin expression is shown as a loading control. (**B**) Relative expression of iNOS and COX-2. Bands on western blots were quantified by densitometry. The values are expressed as the means ± SD of at least three independent experiments (^#^*p* < 0.05 vs. the non-treated control group; *p < 0.05, **p < 0.01 vs. the LPS-treated group).

Western blotting was performed to determine whether the inhibitory effects of CTC on NO and PGE2 production were related to the changes in the expressions of iNOS and COX-2. LPS markedly upregulated iNOS and COX-2 protein levels, and pre-treatment with CTC inhibited these upregulations (Figure 4). These results indicate that the inhibitory effects of CTC on LPS-induced NO and PGE2 production are due to the suppression of iNOS and COX-2. 

### Effects of CTC on the LPS-induced production of TNF-α, IL-1β, and IL-6

**Figure 5:**
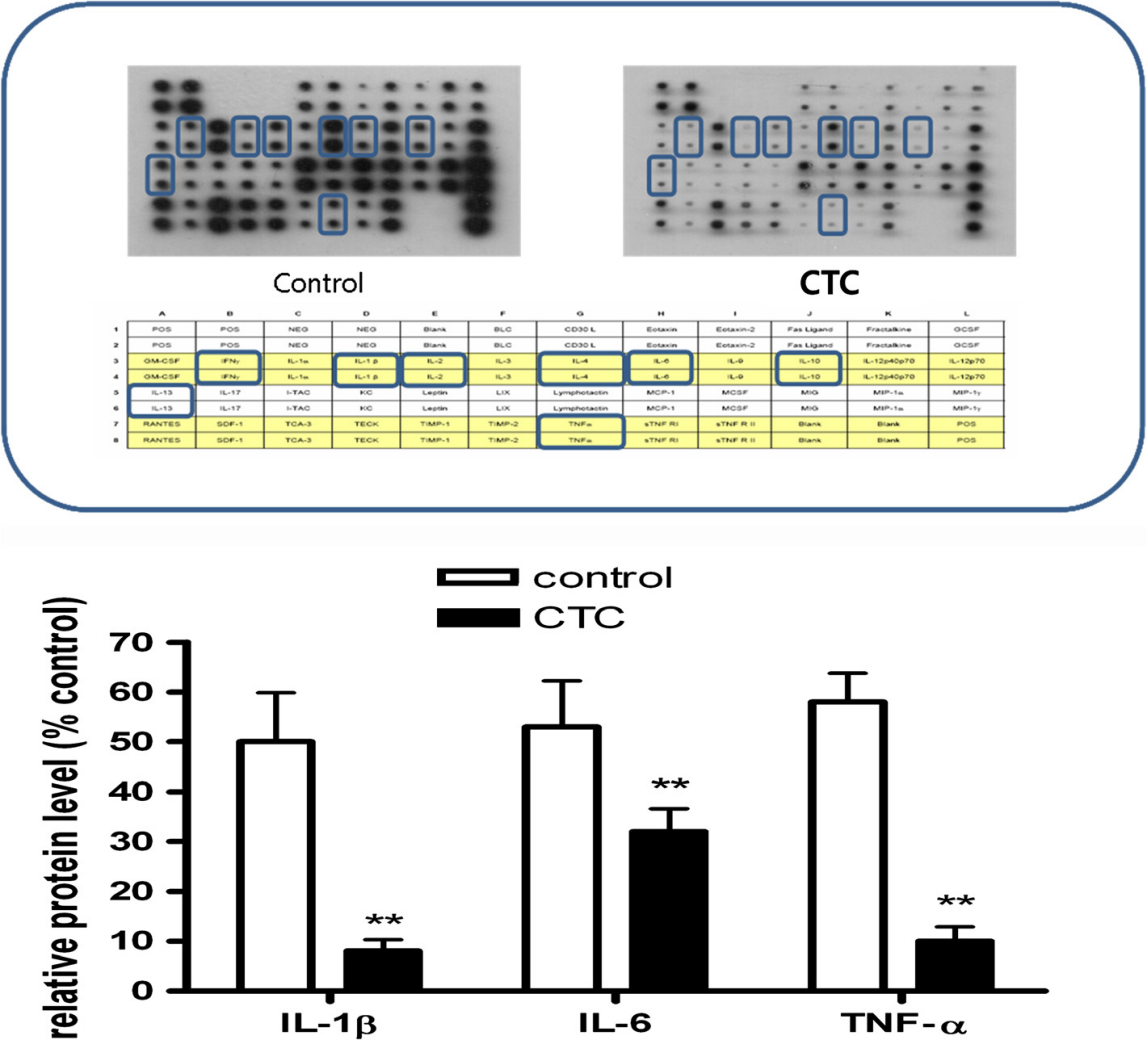
**Effect of CTC on LPS-induced cytokine expression in RAW 264.7 cells. **Levels of the cytokines TNF-α, IL-1β and IL-6 were monitored 24 h after treating cells with LPS (10 μg/ml) with or without CTC.

The effects of CTC on LPS-induced TNF-α, IL-1β, and IL-6 release were investigated using a cytokine array. We observed that pretreatment of RAW 264.7 cells with CTC (250 μg/ml) decreased the TNF-α, IL-1β, and IL-6 production (Figure 5). 

## Discussion

The present study was undertaken to elucidate the pharmacological and biological effects of CTC on the production of inflammatory mediators in RAW 264.7 macrophages. The results indicate that CTC is an effective inhibitor of LPS-induced NO, PGE2, TNF-α, IL-1β, and IL-6 production in these cells. Furthermore, these inhibitory effects of CTC were accompanied by dose-dependent decreases in the protein levels of iNOS and COX-2. These findings indicate that the expression of iNOS and COX-2 in RAW 264.7 cells is suppressed by CTC, and that this suppression leads to the downregulation of NO and PGE2.

NO is a key inflammatory mediator, and excessive NO production occurs in both acute and chronic inflammation. NO is synthesized from l-arginine by NOS isoenzymes, one of which is iNOS, which is expressed predominantly in activated macrophages [17]. Similarly, PGE2 is a well-known inflammatory mediator produced by the action of COX-1 and COX-2 on arachidonic acid. COX-2 expression is induced by cytokines, LPS, and other activators during inflammation, resulting in the release of high levels of PGE2 at the sites of inflammation [18]. In this study, we showed that in LPS-stimulated macrophages, CTC dose-dependently inhibited NO and PGE2 production. The levels of iNOS and COX-2 were also downregulated by CTC, suggesting that this was the mechanism underlying the observed suppression of NO and PGE2 production.

LPS stimulates macrophages to produce iNOS, COX-2 and pro-inflammatory cytokines such as TNF-α, IL-1β, and IL-6 [17]. In addition, these pro-inflammatory mediators play important roles in the pathogenesis of various acute and chronic inflammatory diseases. Thus, blocking the effects of pro-inflammatory mediators could be an effective therapeutic strategy. Inflammatory disorders are characterized, among other events, by the production of significant amounts of free radicals, nitrogen reactive species, and cytokines such as TNF-α, IL-1β, and IL-6 [18]. TNF-α is a critical cytokine in the inflammatory cytokine network; it can induce the release of IL-1β and IL-6, which in turn enhances the sensitivity of the tissue macrophages to TNF-α. TNF-α is an endogenous pyrogen that can cause fever and stimulate the endotheliocytes and leukocytes to release a series of inflammatory mediators (NO, oxyradicals, etc.) that may further promote TNF-α production [19]. TNF-α plays an important role in the pathological damage associated with inflammation. Our results show that CTC significantly suppresses the production of IL-1β, IL-6, and TNF-α, suggesting that CTC exerts its anti-inflammatory effects via inhibition of IL-1β, IL-6, and TNF-α production.

## Conclusions

In summary, the results of the present study show that the anti-inflammatory effects of CTC are achieved via the modulation of macrophage-mediated inflammation-associated functions such as the overproduction of cytokines, NO, and PGE2. Moreover, the described CHCl_3_ fraction was found to be the active fraction in this context.

## Abbreviations

CHCl_3_: Partition of the methanol extract of *Cudrania tricuspidata*; CTC: Lipopolysaccharide; LPS: Prostaglandin E_2_; PGE_2_: Nitric oxide; NO: Inducible isoform of NO synthase; iNOS: Cyclooxygenase-2 COX-2.

## Competing interests

The authors declare that they have no competing interests.

## Authors’ contributions

GY carried out the in vitro studies and drafted the manuscript. GY and ML carried out the immunoassays. HC and IH participated in the design of the study and performed the statistical analysis. KL conceived of the study, and participated in its design and coordination and helped to draft the manuscript. All authors read and approved the final manuscript.

## Pre-publication history

The pre-publication history for this paper can be accessed here:

http://www.biomedcentral.com/1472-6882/12/250/prepub
